# Dramatic increase in lever-pressing activity in rats after training on the progressive ratio schedule of cocaine self-administration

**DOI:** 10.1038/s41598-021-98313-0

**Published:** 2021-10-04

**Authors:** Vladimir L. Tsibulsky, Andrew B. Norman

**Affiliations:** Department of Pharmacology and Systems Physiology, University of Cincinnati College of Medicine, Cincinnati, OH 45267 USA

**Keywords:** Neuroscience, Learning and memory, Classical conditioning

## Abstract

Transition from the highest rate of lever-pressing activity during the unloading (extinction) phase of a cocaine self-administration session to an extremely low activity rate during the remission phase is in many cases gradual. This makes it difficult to assess the duration of the unloading phase after a fixed ratio 1 (FR1) or breakpoint after a progressive-ratio (PR) self-administration session. In addition, 3–5 days of training under the PR schedule results in a dramatic and persistent increase in the rate of presses during PR sessions and in the unloading phase following FR1 self-administration sessions. The goals of this study were to find the definition of the last press demarcating the border between the unloading and remission phases of the session and to determine if this border was also affected by PR training. Rats were trained to self-administer cocaine under the FR1 schedule and then under the PR schedule of drug delivery. Distributions of inter-press intervals (IPIs) during the unloading phase in sessions before and after PR training were compared. It was found that the distribution of cocaine-induced IPIs during the unloading phase was lognormal, bimodal, and independent of previously injected cocaine unit doses. The first mode represented intervals within the short bouts of stereotypic presses and the second mode represented intervals between bouts. The two modes were approximately 0.7 s and 21 s during unloading prior to and 0.6 s and 1.5 s after PR self-administration training. The total number of presses per unloading phase increased eightfold. When the FR1 schedule was restored, the intervals between bouts remained very short for at least 7–10 days and only then started a gradual increase towards baseline levels. The last unloading press was defined as the press followed by the IPI longer than the defined criterion. PR training resulted in a substantial and long-lasting increase in lever-pressing activity during unloading. The duration of the unloading phase did not depend on the rate of lever-pressing activity.

## Introduction

In a typical drug self-administration study, an animal is implanted with an indwelling intravenous catheter and trained to press one of two levers. The rate of acquisition is the highest under the fixed ratio 1 (FR1) schedule when a drug injection follows every lever press except presses during time-out periods. The FR1 schedule is sometimes called a continuous reinforcement schedule and represents the simplest requirement to obtain a drug dose. To the best of our knowledge, there is no published data on effects of the FR schedules higher than FR1 on the rate of acquisition of drug self-administration. However, it was hypothesized that low requirements along with a high reinforcement magnitude^1^ facilitate the acquisition. Some researchers argue that while FR1 is good for training, it is not good for the assessment of motivation to get a reinforcer^2^. A progressive ratio (PR) schedule is a type of intermittent reinforcement schedule when a response ratio requirement gradually increases^3^. The progressive increase of the ratio was proposed to titrate the level of motivation under different conditions^4^.

Training to self-administer drugs under the PR schedule of drug delivery leads to a dramatic increase in lever pressing activity^5^ compared with FR1 self-administration. This effect received very little attention from researchers and perhaps it seems trivial. Indeed, since an experimenter delivers the drug after not one but many responses, animals must increase the number of responses as required. However, surprisingly, they achieve this higher ratio by increasing not the duration of activity but by increasing the rate of activity and that is not required per se by the PR schedule.

The high rate of lever-pressing activity during PR self-administration resembles behavior during the unloading phase (often referred to as the extinction phase) after termination of access to cocaine in FR1 and it is strikingly different from the behavior during maintained FR1 self-administration. The rate of responses during unloading is the highest observed compared to all other phases of an FR1 self-administration session, and this rate does not depend on the cocaine doses previously injected during the maintenance phase^6^. In addition, similar to PR self-administration, the unloading phase also ends by cessation of lever-pressing activity. Therefore, self-administration under the PR schedule maybe viewed as a series of short unloading phases separated by drug injections, i.e., as a mixture of loading and unloading. In contrast to PR self-administration, FR1 self-administration is regular, inter-injection intervals are dose-dependent and maintained for many hours as long as drug is available. Under the PR schedule, inter-injection intervals are also dose-dependent but not regular and increase along with ratio requirements^7^ bringing about cessation of lever-pressing activity called “break point” (BP). It suggests that self-administrations under the FR1 and PR schedules represent qualitatively distinct phases of self-administration sessions.

The lever-pressing rate during the unloading phase is the highest and during the following remission phase (when lever-pressing is extinguished) is the lowest among all phases of self-administration session. However, the transition from the unloading phase to the remission phase of a cocaine self-administration session is not abrupt in many cases. Earlier we developed the criterion for the last press of PR self-administration based on 3-σ rule^8^. It was possible because inter-press intervals (IPIs) before the breakpoint were lognormally distributed. The same rationale could not be applied to define the last press after FR1 self-administration because the probability of longer intervals to occur gradually increases as the unloading phase progresses making the IPI distribution skewed. Therefore, a new approach was tested and a new criterion was developed based on this observation.

Herein we also present the results of statistical analyses of unloading lever-pressing activity in self-administration sessions under the FR1 schedule before and after rats were trained under the PR schedule. The analysis of IPIs under the PR schedule in the same animals was published earlier^8^.

## Methods

### Cocaine self-administration training

Six male Sprague Dawley rats (from Harlan, Indianapolis, IN, initial weight 180–200 g and 400–500 g over the duration of the studies) were housed individually on a 12/12 h light/dark cycle (lights on at 6:00 a.m.) and food and water were available *ad lib*. Herein we present results obtained from the same experiment as in our previous publication^8^ but the data set was differently analyzed.

Indwelling catheters were surgically implanted into the right jugular vein under halothane anesthesia. Beginning five or seven days after the surgery, rats were trained to self-administer cocaine HCl using the FR1 schedule with a time-out (TO) period equal to the injection duration but not shorter than 5 s. The house light in the chamber was turned on during the time of pump activation (5–40 s) signaling TO. Training at the unit dose of 1.5 μmol/kg (0.51 mg/kg) cocaine continued until individual rats met the criterion of stable maintained self-administration. This criterion was no significant change of the mean and standard deviation of the inter-injection intervals between five consecutive sessions. At the same time the proportion of inactive lever presses was lower than 2.5% of the total presses.

Cocaine unit doses were controlled by the duration of pump activation. After self-administration of the initial priming (programmed escalating doses until initiation of self-administration or 2–4 doses fixed at 1.5 μmol/kg) and loading phases of the session, the program allowed self-administration of 50 doses of 0.3 μmol/kg under FR1 and then switched to PR schedule delivering one of the following doses: 0.75, 1.5, 3.0, 6.0 or 12.0 μmol/kg. These doses were selected in pseudo random fashion between sessions. Every cocaine unit dose was repeated 5 times totaling 150 sessions from 6 rats. Data from 24 sessions were excluded from analyses because animals did not show normal patterns of self-administration (6 sessions), due to technical errors (3 sessions) or because BPs in first 1–2 sessions under the PR schedule were substantially lower than after that (15 sessions). Results of all individual sessions before exclusions can be found in Supplementary Materials.

The total number of injections at each cocaine dose delivered during self-administration sessions maintained under the FR1 schedule was preset to keep the total duration of self-administration between 2 and 3 h. Sessions were aborted any time after the first inter-press interval longer than 30 min. This criterion is consistent with the conventional 1 h since the last drug delivery, as lever-pressing activity during extinction was observed for approximately 30 min.

### Hardware and software

Test chambers (modified chambers from Lafayette Instrument Co., Lafayette, IN) were each equipped with an active and an inactive lever. Each chamber was situated inside of a laminated wooden compartment (43 × 61 × 35 cm) that provided sound attenuation and was equipped with a house light (7-W fluorescent bulb). Syringe pumps (model PHM-100, Med Associates Inc., Georgia, VT) were situated outside of the laminated compartments. Computers controlled conditioned (light) and unconditioned (drug injection) stimuli using a program written in MedState Notation language (Med Associates, Inc., VT). At every lever-press, the time and the current calculated cocaine level in the body were recorded^9^.

### Catheter patency control

In order to maintain catheter patency, after every session the catheter was flushed with 0.2–0.5 ml of sterile saline containing 10 units/mL heparin. Catheter patency was evaluated by i.v. administration of short acting barbiturate methohexital (Brevital 6 mg/kg, 3 s injection). The catheter was considered patent if administration of Brevital produced a loss of righting reflex within 5 s after the injection was completed and the latency to the recovery of the righting reflex was greater than 100 s. A failed catheter was removed and a new catheter was implanted into the same right or into the left jugular vein. Self-administration data from each rat were gathered as long as 7.8 ± 0.6 months (mean ± SEM).

### Experimental design

Three series of self-administration sessions were as follows: Baseline FR1 sessions, PR sessions, and Post-PR FR1 sessions.

During the first part of the experiment, after training was over, rats were placed in the chambers and from the beginning of a session received cocaine injections under the FR1 schedule (Baseline FR1 sessions). The first maintenance dose of cocaine was selected from 0.3, 0.75 and 1.5 µmol/kg and the second dose was selected from the range of 3.0, 6.0 and 12.0 µmol/kg in pseudo-random order. The number of injections at each unit dose was preset in order to keep the total self-administration duration at any dose around 1 h totaling approximately 2 h. After the last injection, the syringe pump was inactivated and the unloading phase of the session continued until the first post-press interval longer than 30 min was observed.

The next 25–27 sessions started with self-administration of 50 cocaine unit doses of 0.3 µmol/kg under the FR1 schedule and then continued with self-administration under the PR schedule (PR sessions). Under the PR schedule, the unit dose was randomly selected from the following: 0.75, 1.5, 3, 6 or 12 μmol/kg. The session was terminated at any time after 30 min period without lever presses.

During the final phase of the experiment (Post-PR FR1 sessions), sessions started with a series of non-contingent presentations of the cue signal light at variable intervals of 100–600 s. Initially these signals occasionally induced some lever-presses. Thirty minutes after the last lever press, the catheter was automatically filled with the cocaine solution and non-contingent injections of escalating doses of cocaine were administered every minute until the peak cocaine level reached 6.5 µmol/kg. Then programmed injections were stopped but the session continued and rats started self-administration of cocaine under the FR1, TO5-40 s schedule for approximately 2 h exactly as in the Baseline FR1 sessions. The syringe pump was then disconnected and the session was terminated sometime after 30 min without lever presses.

### Progressive ratio function and BP measurements

For the sake of convenience, the relationship between the number of injections and the number of presses required to receive them was expressed in the form of the discrete function called progressive ratio function or *R* = *f*(*n*) where *R* is the ratio and *n* is a number of injections. The following equation for PR increments was selected (similar to that proposed by Roberts and Richardson^10^):

1 $${\text{Ratio }} = {\text{ truncate }}\left( {{5}\cdot\left( {{\text{exp}}\left( {j\cdot\left( {n{-}{ 1}} \right)} \right) \, {-}{ 1}} \right)} \right) \, + { 1}$$

where the slope factor *j* = 0.4 was generating ratios: 1, 3, 7, 12, 20, 32, 51, 78, 118, 178, 268, 403, 603, 902, 1348, 2013. In this study, the total number of delivered injections under a PR schedule according to Eq. (1) never exceeded 16.

### Survival analysis

The transition from unloading to remission is not abrupt meaning that during the unloading phase the probability to observe longer and longer IPIs gradually increases over time. We assume that there is an IPI which might be used to demarcate the border between the unloading and remission phases of the session. Survival analysis would be a suitable method to find this critical IPI. Survival analysis is applied when an event terminates the existence of something. In this study, we analyzed the survival of the unloading phase characterized by short IPIs. The “death” of the unloading phase was declared after occurrence of the first critically long IPI. This critical event, the first IPI ≥ criterion, was determined in the following way. First, we selected a series of arbitrary preset IPI values, called putative remission criteria: 100, 300, 450, 600, 900, and 1800s. The range of putative criteria was determined by the a priori selected criterion of 30 min (1800s) without an active lever press meaning that the session was terminated any time after the first IPI ≥ 1800s. Then, the mean time of the unloading phase elapsed from the first unloading press to the press followed by this critical IPI was determined for every session using every putative criterion. An effective duration of the unloading phase (ED_50_, the mean unloading time elapsed to the point when 50% of sessions reached the remission criterion) was determined for each criterion and for each rat. Finally, the averaged between rats ED_50_ was plotted as a function of the corresponding putative criteria.

One should bear in mind that the value of the remission criterion depends on the distribution of IPIs during the unloading phase of the session and cannot be determined for the sessions under PR schedule of drug delivery. This is because in PR sessions the unloading phase was interrupted by loading injections. This means that the same survival analyses could not be applied to PR sessions and was applied only to Baseline FR1 and Post-PR FR1 sessions.

### Statistical analysis

All statistical (Pearson linear correlation, nonlinear regression and survival analyses) and visual analyses of data were conducted using SigmaPlot software v. 14.0 (Systat Software, Palo Alto, CA, https://systatsoftware.com). The distinct phases of the self-administration session were differentiated based on changes in IPIs. Paired *t*-test and the two-way ANOVA were used to test for a significant difference with the significance criterion set at *α* = 0.05. Differences with *p* ≤ 0.005 were expected to be reproducible in more than 80% of replications.

### Drugs

Cocaine HCl was obtained from Research Triangle Institute (Research Triangle Park, NC) under the National Institute on Drug Abuse Drug Supply Program. The drug was dissolved in saline containing one unit/ml of heparin (American Pharmaceutical Partners, Inc., Schaumburg, IL) and then passed through a sterilizing 0.2 μm acetate filter immediately prior to use in the self-administration studies. The concentration of cocaine solution was 40 μmol/mL. Methohexital sodium (Brevital, Jones Pharma, St. Louis, MO) was used as 1% (w/v, 0.5 mL/kg) solution in normal saline was prepared immediately before injections.

## Results

### Inter-injection intervals during maintenance

The first maintenance phase of every session started with self-administration under the FR1 schedule. The two-way ANOVA of the inter-injection intervals during maintained self-administration at the cocaine unit dose of 0.3 µmol/kg showed significant difference between individual rats (F_5,314_ = 4.598, *p* < 0.001) but not significant difference between three series of sessions (F_2,314_ = 1.597, *p* = 0.204). For Baseline FR1 sessions, PR sessions and Post-PR FR1 sessions, the mean ± SEM inter-injection intervals were 56.2 ± 2.8 s, 54.4 ± 2.3 s, and 53.5 ± 2.4 s, respectively.

### Inter-press intervals during unloading

The distribution analysis demonstrated that for all sessions IPIs during unloading after FR1 self-administration were lognormally distributed and had two modes. Therefore, all statistical analyses were conducted using logarithmically transformed IPI values. There was a significant correlation between the two peaks during Baseline FR1 sessions (*r* = 0.417, *p* = 0.022, *n* = 30). This correlation strengthened during Post-PR FR1 sessions (*r* = 0.744, *p* < 0.001, *n* = 30). The two-way ANOVA showed that the cocaine unit dose did not affect the distribution of IPIs (the biggest effect of a dose was on the second peak in Post-PR FR1 sessions, F_4,29_ = 2.530, *p* = 0.073), therefore data were collapsed across cocaine unit doses (Table 1). In contrast, both peaks of IPI distribution demonstrated significant individual variability during the unloading phase in Post-PR FR1 sessions (F_4,29_ = 10.052, *p* < 0.001 and F_4,29_ = 9.294, *p* < 0.001 for peaks 1 and 2, respectively). IPI distributions in individual rats can be found in Supplementary Materials.

**Table 1:** Peaks of the lognormal distributions of IPIs during unloading phases of cocaine self-administration sessions under the FR1 schedule before and after sessions under the PR schedule in individual rats.

Rat #	Baseline FR1 sessions		Post-PR FR1 sessions	
	Peak 1*	Peak 2	Peak 1	Peak 2
1	0.76 (0.68–0.85)	34.4 (16.1–73.3)	0.54 (0.54–0.55)	0.79 (0.68–0.91)
2	0.96 (0.94–0.98)	31.7 (23.6–42.7)	0.60 (0.58–0.63)	1.40 (1.27–1.54)
3	0.68 (0.64–0.72)	55.3 (45.2–67.6)	0.74 (0.71–0.77)	1.36 (1.15–1.61)
4	0.69 (0.61–0.77)	11.0 (7.15–17.0)	0.65 (0.61–0.69)	1.95 (1.66–2.29)
5	0.80 (0.69–0.92)	47.1 (33.4–66.5)	0.89 (0.85–0.94)	4.73 (2.91–7.69)
6	0.72 (0.63–0.83)	22.9 (14.9–35.3)	0.41 (0.34–0.50)	0.78 (0.63–0.96)
Mean	0.68 (0.62–0.75)	21.2 (15.4–29.2)	0.62 (0.56–0.69)	1.49 (1.15–1.94)

*Peaks represent the most frequent IPIs in seconds for both modes of the distribution. The range of standard errors is in brackets.

**Figure 1:**
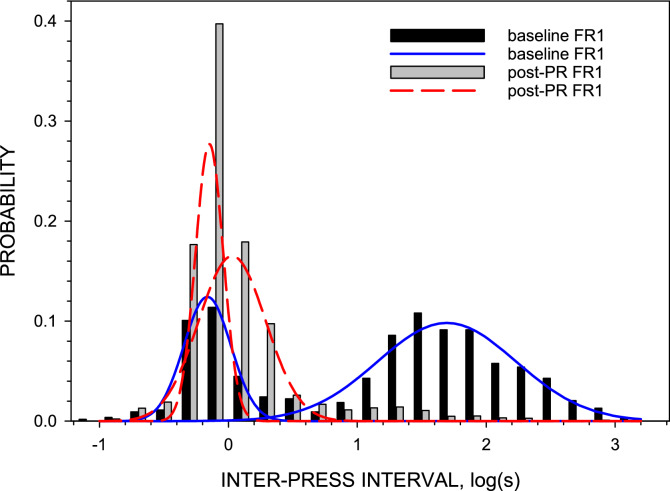
Probability density functions of IPIs during unloading phase in Baseline FR1 and Post-PR FR1 sessions in a representative rat. Both distributions were lognormal and bimodal. Lines represent the best fit of non-linear regression analysis before (solid blue lines) and after (long-dashed red lines) PR sessions. SigmaPlot v. 14.0 (https://systatsoftware.com).

Comparison between Baseline FR1 and Post-PR FR1 sessions did not reveal any significant correlation between corresponding peaks but showed that after PR sessions both peaks shifted to the left (Table 1, Fig. 1). The mean peak of short IPIs changed slightly from 0.68 s to 0.62 s (paired *t* = 0.526, *p* = 0.62, *n* = 6). The most dramatic 14-fold decrease was observed in the mean peak of long IPIs from 21.2 s to 1.5 s (paired *t* = 7.40, *p* = 0.0007). This increase in the rate of presses was sustained for at least 7–10 days after the last PR schedule session and then started to gradually decrease towards baseline levels.

The total number of unloading presses (mean ± SEM) during Baseline FR1 sessions was significantly lower than the total number of unloading presses during Post-PR FR1 sessions (41.8 ± 5.7 *vs* 376.5 ± 67.3, paired *t* = 5.294, *p* = 0.003, *n* = 6). The total number of presses after the last injection of the highest cocaine unit dose of 12 μmol/kg (4.08 mg/kg) during PR sessions (317.1 ± 55.9 presses, data from^8^) was also significantly higher than unloading Baseline FR1 session presses (paired *t* = 5.504, *p* = 0.003). However, the difference between PR sessions and Post-PR FR1 sessions was not statistically significant (317.1 *vs* 376.5 presses, paired *t* = 2.169, *p* = 0.082). Cross correlation analysis within subjects showed that the only significant correlation was between the number of presses during PR and Post-PR FR1 sessions (*r* = 0.918, *p* = 0.01, *n* = 6).

### Survival analysis of inter-press intervals

**Figure 2:**
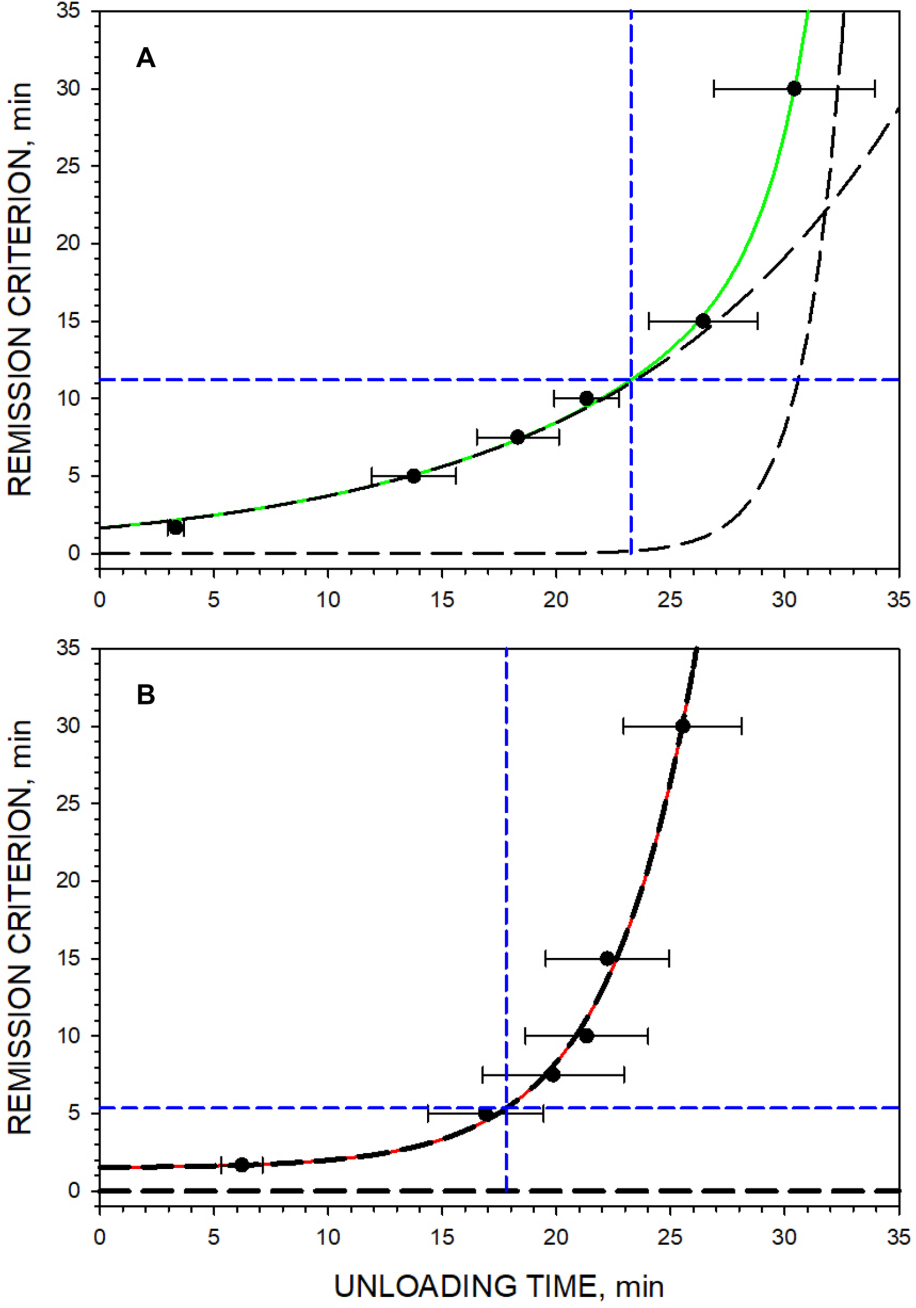
Survival analyses of long IPIs as a function of elapsed unloading time during Baseline FR1 sessions (Panel **A**) and during Post-PR FR1 sessions (Panel **B**). Symbols represent the mean ± SEM unloading duration (ED_50_) in 6 rats at which the first preset long interval occurs (see *y*-axis). The solid lines represent the best fit of the nonlinear regression analysis according to *y* = *y*_0_ + *a*·exp(*b·x*) + *c*·exp(*d·x*). In panel (**A**), solid green line: *y*_0_ = 5.14∙e^–9^, *a* = 1.65, *b* = 0.08, *c* = 3.43∙e^–7^ and *d* = 0.57. In panel (**B**), solid red line: *y*_0_ = 1.49, *a* = 1.65, *b* = 0.26, *c* = 0.00 and *d* = 0.00. Long dash lines (bold) represent the two exponential components separately. The horizontal short dash lines represent the best remission criteria estimates of 11.22 min (673 s; panel **A**) and 5.35 min (322 s; panel **B**) when the slope of solid lines, the first derivative, was unity: *y´* = *a·b·*exp(*b·x*) + *c·d·*exp(*d·x*) = 1. The vertical short dash lines represent the border between slow and fast raising parts of the function at the elapsed unloading time (Panel **A**: 23.26 ± 2.69 min; Panel **B**: 17.80 ± 2.72 min) dividing unloading and remission phases. SigmaPlot v. 14.0 (https://systatsoftware.com).

Survival analysis was conducted to determine a criterion for the last unloading press in Baseline FR1 sessions. The mean time elapsed after the beginning of the unloading phase until the first IPI of equal or longer duration than the preset value (called the putative remission criterion) was plotted against this preset value (Fig. 2A). The regression analyses showed that the bi-exponential growth function *y* = *y*_0_ + *a*·exp(*b·x*) + *c*·exp(*d·x*) provides a very good fit to the experimental data. Therefore, the first derivative of the survival function is also an exponential function of this kind *y´* = *a·b·*exp(*b·x*) + *c·d·*exp(*d·x*). This means that there is a particular point on the *x*-axis when the slope (*y´*) of the survival curve is unity. The value of *x* at *y´* = 1 was found by a numerical method because there is no analytical solution for equations of this kind. The *y*-value at which the first derivative is *y´* = 1 may be justified as the remission criterion because after that point every additional second of the session results in more than one second added to the IPI. The average IPI demarcating the end of the unloading phase of the Baseline FR1 sessions was 673 s (11.22 min). This means that the last press of the unloading phase, which is at the same time the first press of the remission phase, was defined as the press followed by the IPI ≥ 11.22 min. This press occurred 23.26 ± 2.69 min after the beginning of the unloading phase (Fig. 2A).

The higher number of unloading lever presses during the Post-PR FR1 sessions suggested that the remission criterion might be changed in rats experienced with self-administration under the PR schedule. The same survival analysis was repeated in order to find the remission criterion in Post-PR FR1 sessions. Indeed, the survival analysis of IPIs showed that one of the two exponents was almost gone and a mono-exponential function provided a good fit to the experimental data (Fig. 2B). The average IPI indicating the end of the unloading phase of the Baseline FR1 sessions was 321 s (5.35 min). The remission criterion became significantly shorter (5.35 min *vs* 11.22 min, paired *t* = 2.11, *p* = 0.03) in rats after PR training.

### Duration of the unloading phase

**Figure 3:**
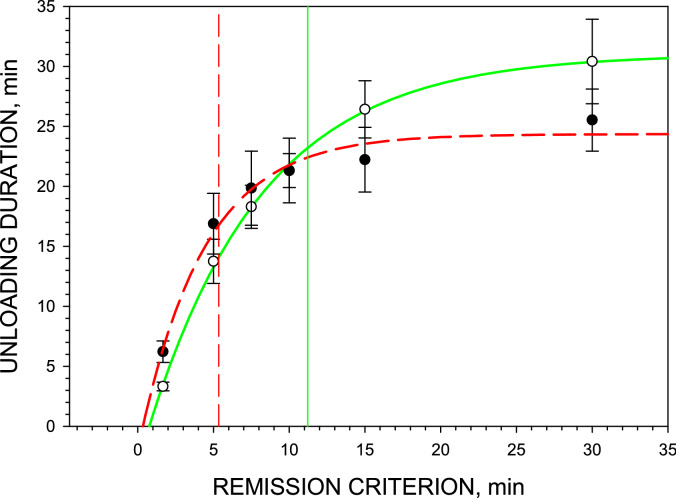
Unloading duration as a function of the putative remission criterion. Symbols represent the mean ± SEM time elapsed between the first and the last unloading presses in Baseline FR1 sessions (○) and in Post-PR FR1 sessions (●) in 6 rats. Curved lines represent the best fit of the nonlinear regression analyses according to *y* = *y*_0_ + *a*∙[1–exp(– *b*∙x)] where *y*_0_ = 3.3, *a* = 34.3, *b* = – 0.13 for Baseline FR1 (solid green line) and *y*_0_ = 2.0, *a* = 26.4, *b* = – 0.23 for Post-PR FR1 sessions (dashed red line). Vertical lines show the projection of the corresponding remission criteria on the unloading duration curves. SigmaPlot v. 14.0 (https://systatsoftware.com).

As expected, the unloading duration was proportional to the putative remission criterion (F_5,11_ = 21.367, *p* = 0.002), confirming the initial observation that longer IPIs occur later in the unloading phase (Fig. 3).The two-way ANOVA of the time elapsed between the first and the last unloading presses showed that there is no difference between the Baseline FR1 and Post-PR FR1 sessions at any putative remission criterion (F_1,11_ = 0.0295, *p* = 0.870).

There was no significant effect of cocaine unit dose on unloading durations, local rates (*LR*, *i.e*. between penultimate and ultimate injections at all cocaine unit doses), terminal local rates (*LR*_*term*_, *i.e*., after the last injection of 6 or 12 µmol/kg) in PR sessions and final local rates (during the entire unloading phase) in FR1 sessions. Therefore, data were collapsed across all doses and the mean values were calculated for each individual rat (Table 2). In PR sessions, cocaine levels after the last self-administered cocaine dose of 6 or 12 µmol/kg decreased from the satiety threshold to the remission threshold exactly as they did in FR1 sessions. There was no statistical difference between both unloading durations and *LR*_*term*_ at these two doses. Therefore, the duration and lever-pressing activity were collapsed across these two doses and compared with the corresponding parameters of the Baseline FR1 and Post-PR FR1 sessions (Table 2). Duration of unloading phases were determined in the Baseline FR1, PR and Post-PR FR1 sessions using the corresponding remission criteria (11.2, 7.5 and 5.4 min). The remission criterion for PR sessions was published earlier^8^. Multiple linear regression analyses showed no significant correlation between unloading durations or between unloading *LR*_*term*_ in three types of sessions. Analyses showed that PR training did not affect unloading duration compared with the Baseline FR1.

**Table 2:** Duration of unloading phases, *LR*_*term*_ and *LR* of lever-pressing activity in individual rats.

Rat #	Baseline FR1 sessions		PR sessions*			Post-PR FR1 sessions	
	Unloading duration (min)	Local rate *LR*_*term*_ (press/min)	Unloading duration (min)	Local rate *LR* (press/min)	Local rate *LR*_*term*_ (press/min)	Unloading duration (min)	Local rate *LR*_*term*_ (press/min)
1	27.2 ± 2.4	1.31 ± 0.29	13.4 ± 2.2	49.3 ± 9.6	20.8 ± 2.9	18.8 ± 1.8	22.5 ± 2.9
2	14.5 ± 2.3	1.75 ± 0.21	21.5 ± 3.3	22.9 ± 5.3	10.2 ± 1.8	21.7 ± 1.2	16.2 ± 1.6
3	22.7 ± 2.1	0.98 ± 0.08	18.5 ± 2.5	25.5 ± 6.0	16.4 ± 4.6	17.8 ± 1.5	15.3 ± 2.4
4	27.5 ± 1.4	1.83 ± 0.14	23.6 ± 1.7	36.4 ± 9.6	17.9 ± 2.5	28.6 ± 2.0	14.9 ± 2.1
5	20.2 ± 1.3	1.27 ± 0.09	10.6 ± 1.8	15.8 ± 3.4	10.4 ± 2.2	16.2 ± 2.0	8.2 ± 0.9
6	25.5 ± 2.4	1.34 ± 0.32	22.0 ± 1.0	51.9 ± 13.3	23.5 ± 4.9	17.7 ± 2.0	26.7 ± 4.2
Mean	22.9 ± 2.0	1.38 ± 0.14	18.3 ± 2.1	33.6 ± 6.0	16.5 ± 2.2	20.1 ± 1.9	17.3 ± 2.6

*—data collapsed across cocaine unit doses of 6 and 12 µmol/kg.

The overall rate of lever-pressing activity (*LR*_*term*_) during the unloading phase after PR training increased by 12-fold (paired *t* = 5.88, *p* = 0.002) and remained high in Post-PR FR1 sessions (paired *t* = 5.98, *p* = 0.002). The rate of presses before the last cocaine injection under PR schedule, *LR*, was twice as high (paired *t* = 4.28, *p* = 0.008) compared with the activity after the last injection, *LR*_*term*_ (Table 2). There was a significant correlation between *LR* and *LR*_*term*_ (*r* = 0.916, *p* = 0.01) during PR sessions.

### Survival analysis of remission criteria

**Figure 4:**
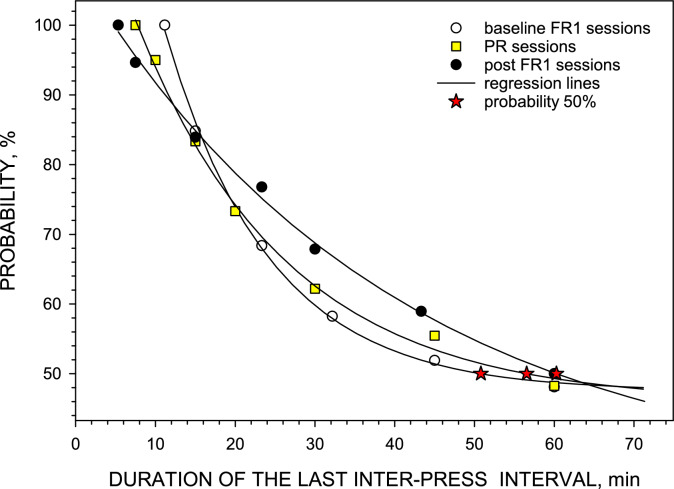
Survival analysis of the IPI after the last press defined according to the remission criteria for Baseline FR1 (11.2 min), PR sessions (7.5 min) and Post-PR FR1 (5.4 min). Symbols represent the time elapsed between the last unloading presses and the first remission press in Baseline FR1 sessions (○), PR sessions (yellow square) and Post-PR FR1 sessions (●) in 6 rats. Lines represent the best fit of the nonlinear regression analyses according to *y* = *y*_0_ + *a*∙exp(–*b*∙*x*) where *y*_0_ = 47.5, *a* = 121.3, *b* = 0.08 for Baseline FR1, *y*_0_ = 45.9, *a* = 81.6, *b* = 0.05 for PR sessions and *y*_0_ = 33.7, *a* = 74.7, *b* = 0.03 for Post-PR FR1 sessions. Stars represent the 50% probability of that the first IPI ≥ remission criterion will be also longer than the following values: 50.8, 56.5 and 60.3 min for Baseline FR1, PR and Post-PR FR1 sessions, respectively. SigmaPlot v. 14.0 (https://systatsoftware.com).

A large number of sessions were terminated when the time elapsed after the last press exceeded 1 h making the survival analyses of the last interval possible. The probability of the first IPI ≥ remission criterion to last up to 1 h and longer was assessed. Figure 4 presents the results of this survival analysis. During Baseline FR1 sessions, the remission criterion was 11.22 min but in 50% of sessions this same interval was, in fact, at least 50.8 min. During Post-PR FR1 sessions, the criterion was 5.35 min but in 50% of cases this same interval was at least 60.3 min. The remission criterion for PR sessions, determined using a completely different method^8^, was 7.5 min but in 50% of cases this same interval was at least 56.5 min and longer.

## Discussion

### IPI distribution

Following an FR1 schedule of drug delivery, IPIs after the termination of access to cocaine showed a lognormal and bimodal distribution. The same kind of distribution was found during cocaine self-administration under the PR schedule of drug delivery^8^ and during extinction of food-induced presses (see review^11^). Distribution parameters did not depend on a cocaine unit dose of a prior self-administration phase under the FR1 nor PR schedules. We hypothesize that very short IPIs of the first mode of the distribution (in the range of 0.6–1.0 s) are under the control of mechanisms regulating cocaine-induced stereotypic behavior. The second mode of the distribution represents periods of inactivity (IPIs longer than 1 s with peaks in the range of 11–55 s) observed between stereotypic bouts (trains of presses with short intervals).

Both peaks gradually shifted to the left during the first 1–3 sessions under the PR schedule. The second peak demonstrated the most dramatic shift to the left after the first and second PR sessions. This resulted in the 14-fold increase in overall lever-pressing activity. After switching back to the FR1 schedule, this effect of the PR schedule on the behavior was sustained for a relatively long period of time (7–10 days) and then started a gradual return back to the Baseline FR1 values. The experiment was not designed to determine the recovery rate but the results in some animals suggest that the process of the gradual decease in lever-pressing rate can take a few weeks.

The rate of lever presses during the unloading phase is the highest among all phases of a self-administration session. It is surprising that the phenomenon of sustainable and more than 12-fold increase in the rate of lever presses during self-administration under the PR schedule did not generate more interest in the field, given its importance in measuring motivation for drugs of abuse. This effect was very robust and was revealed in a very small group of animals (*n* = 6) with p-values below 0.005 which correspond to reproducibility level of 80% and more. It remains unclear, however, if the same increase in lever-pressing rate could be found after training under the PR schedule of other drugs of abuse, or in female rats compared to male rats. This study is the first comparative analysis of the IPI distributions under different schedules of drug self-administration. Further studies are needed to determine gender differences in the effect of PR training on the rate of lever presses, if this effect can be detected during self-administration of other classes of drugs of abuse, and also its reproducibility in larger data sets.

This PR schedule-induced rate increase should not be confused with so-called “incubation of craving” phenomenon^12^ because the former is related to drug-induced presses and the latter is related to cue-induced presses. The PR-induced dramatic increase in unloading lever-pressing activity is rather similar to the schedule-induced polydipsia^13^ or other behavior changes induced by the delay of food delivery^14^.

### Behavior change

We hypothesize that the increase in the lever-pressing rate maybe a result of classical associative learning that takes place during the few first PR sessions. During the initial FR1 self-administration training sessions, animals acquire a new behavioral response induced by increasing cocaine concentrations. This response is a single lever press under the FR1 schedule of delivery. The lever-pressing behavior quickly becomes highly stereotypic and individual rats perform it sometimes in a very different manner. All those presses (the priming effect of cocaine) occur when cocaine levels are at or slightly below the satiety threshold. Very quickly after the beginning of injection, cocaine levels rising above the satiety threshold suppress the same lever-pressing behavior (the satiety effect of cocaine^15^).

Under the PR schedule, the cocaine level continues to fall below the satiety threshold while rats continue to press under higher ratio requirements. Therefore, these cocaine levels lower than the satiety threshold, in contrast to suppression of presses by higher cocaine levels under the FR1 schedule, induce a series of presses between injections. During a couple of first self-administration sessions under the PR schedule, animals acquire a new response that is not a single press but a series (train) of lever-presses. Since this new behavioral response is prepared by the initial training, the learning is complete in 1–3 PR sessions instead of 4–6 sessions typically needed for initial FR1 training. During the first FR1 self-administration sessions, inter-injection intervals become shorter. During the first PR self-administration sessions, inter-bout intervals become shorter and BP becomes higher^16^.

As the PR schedule belongs to the type of intermittent reinforcement (in contrast to continuous reinforcement like FR1), the acquired behavior is very resistant to extinction^17^. That is consistent with the observed persistence of a high lever-pressing rate during 7–10 Post-PR FR1 sessions.

### Relationship between presses and the compulsion zone width

It is well known that cocaine activates dopaminergic mechanisms underlying stereotypy^18^. Lever-pressing behavior as a form of stereotypy, associated previously with cocaine injections, is induced only when the cocaine level is within a certain range called the compulsion zone^19^. During the unloading phase, stereotypic lever presses are observed while cocaine concentrations transit through the compulsion zone from the satiety to the remission threshold. The duration of the unloading phase, therefore, should depend on the two threshold levels and on the cocaine half-life but not on the lever-pressing rate. The inter-injection intervals at 0.3 μmol/kg cocaine unit dose during the first maintenance FR1 phase of all sessions were not changed by PR training, suggesting that cocaine quasi-steady state and, therefore, the satiety threshold were not changed. Furthermore, the duration of the unloading phase did not change suggesting that the remission threshold should also be constant. This is consistent with results of analyses of calculated cocaine level at the satiety and remission thresholds during and after PR sessions (data not shown and will be presented elsewhere).

### Remission criterion

The probability of long IPIs to occur increases dramatically when the falling cocaine level approaches the remission threshold. This observation served as a rationale to define the last press during cocaine self-administration under the PR schedule^8^. Now the same rationale, although using a very different method, was applied to find the remission criterion under the FR1 schedule, i.e., the critical value of the IPI demarcating the time when the unloading phase ends and the remission phase begins. Herein, this time was defined as a moment after which an additional second of elapsed session duration translates in more than an additional second of the probable IPI duration. In other words, the unloading phase of the session ends when the rate at which IPI duration increases is equal and then exceeds the rate at which session duration increases. There is only one point in time when these two rates are equal because the first is growing exponentially and the second is growing linearly.

The above definition of the critical IPI is an operational definition of the border between the unloading and remission phases. During the unloading phase, cocaine level falls down from the satiety threshold to the remission threshold. At the cocaine remission threshold or below, cocaine does not induce stereotypic lever presses. This is a mechanistic definition of the border between the unloading and remission phases. The relationship between these two definitions of the remission threshold across all three types of sessions will be presented somewhere else (data not shown).

The values of the proposed criteria of the beginning of the remission phase are relatively short (5 ≤ IPI ≤ 12 min). However, the survival analysis of these critical IPIs demonstrates that in 50% of sessions these IPIs are in fact longer than 50–60 min. Therefore, values for critical IPIs reported herein are robust and should provide similar results as analyses of FR1 and PR self-administration in studies with arbitrary long criterion of 60 min after the last injection.

## Conclusions

Training under the PR schedule of cocaine delivery produced sustainable 12-fold increase in the overall rate of unloading presses primarily due to shortening the intervals between bouts. The last unloading press was defined as the press followed by the IPI longer than the defined criterion (11.22 min in this study). PR training significantly shortened the IPI criterion of the remission phase in this study from 11.2 min in Baseline FR1 sessions and to 5.4 min in Post-PR FR1 sessions while the decrease in duration of the unloading phase was not statistically significant.

## Supplementary Information

Supplementary Tables.

Supplementary Figures.
