# Supplementary material for: Dramatic increase in lever-pressing activity in rats after training on the progressive ratio schedule of cocaine self-administration
Source: Sci Rep. 2021 Oct 4;11:19656. doi: 10.1038/s41598-021-98313-0 (PMC8490463; doi:10.1038/s41598-021-98313-0)

RAT 387

Baseline FR1

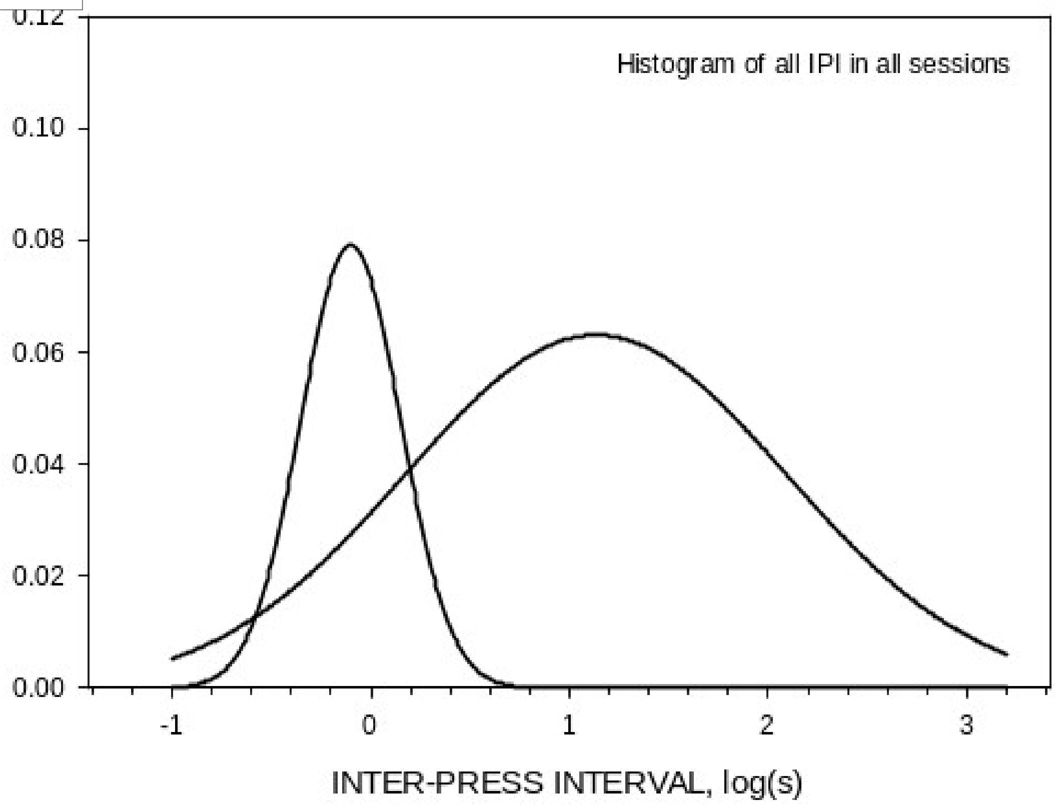

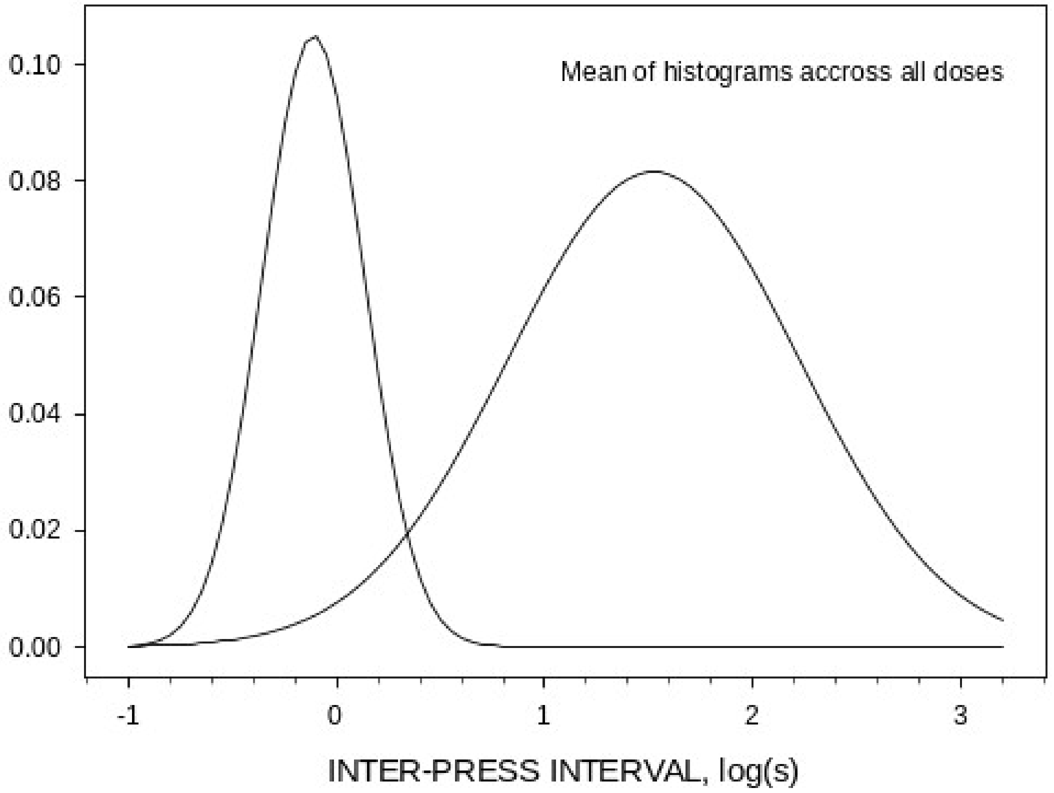

RAT 387

Post PR FR1

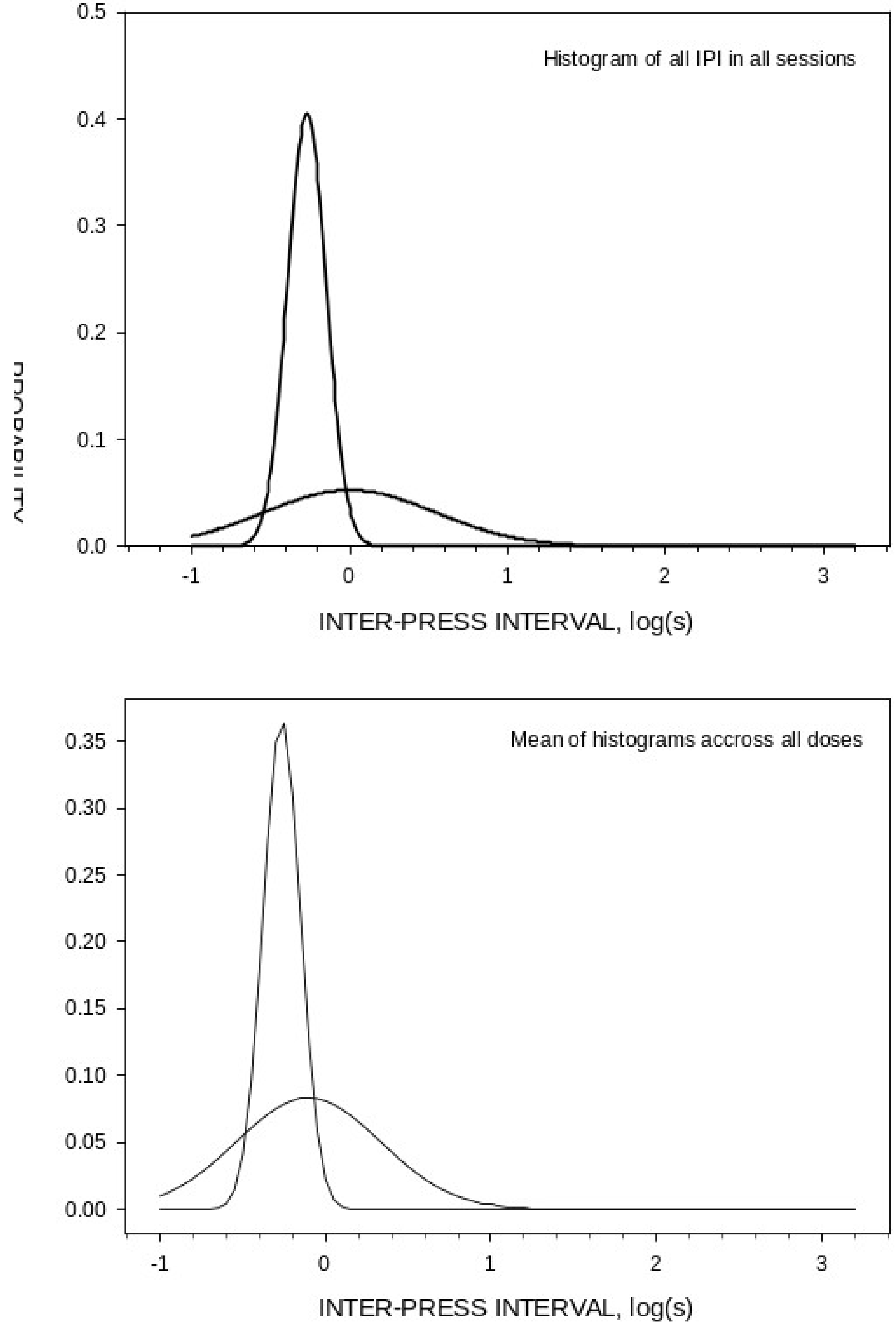

RAT 390

Baseline FR1

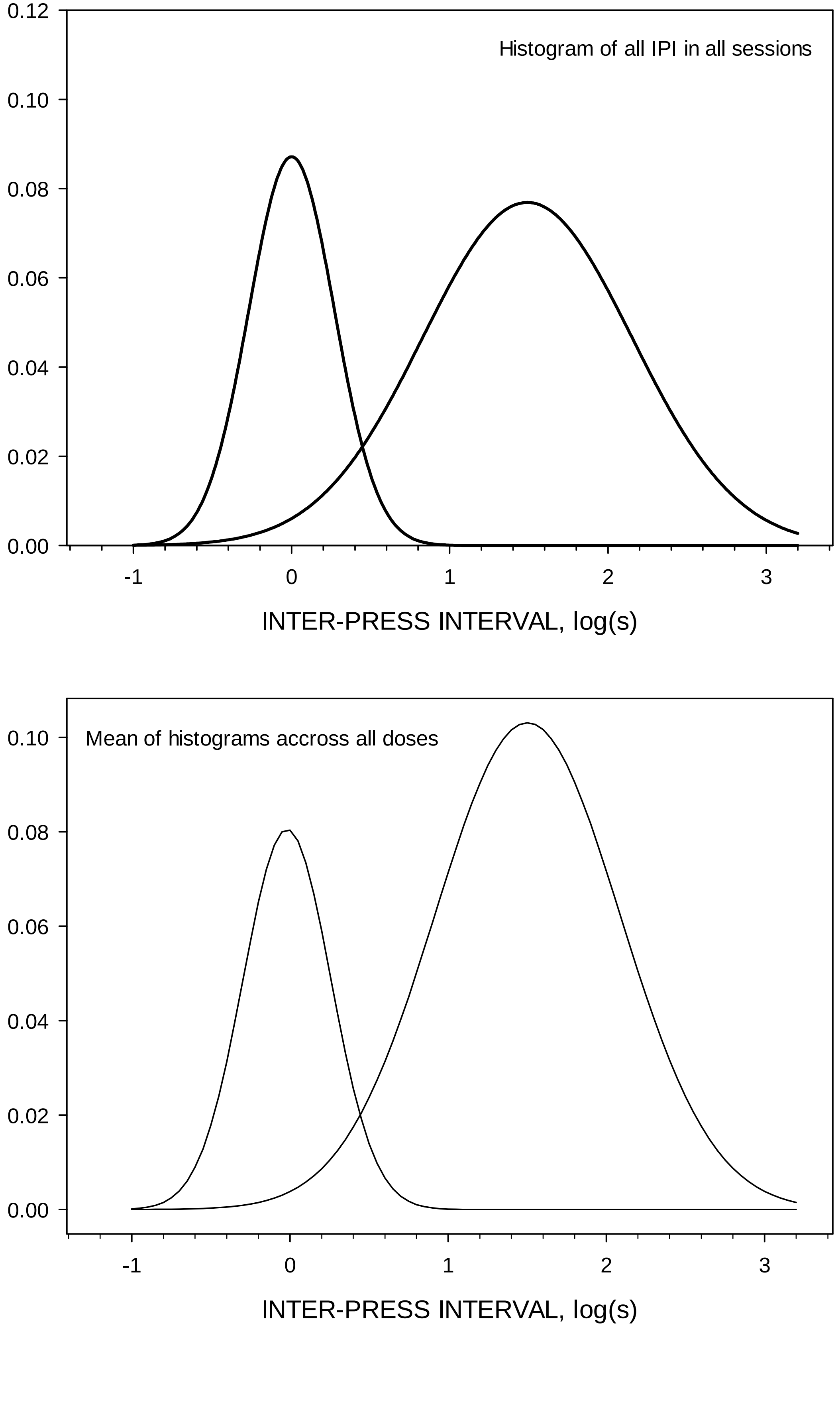

RAT 390

Post PR FR1

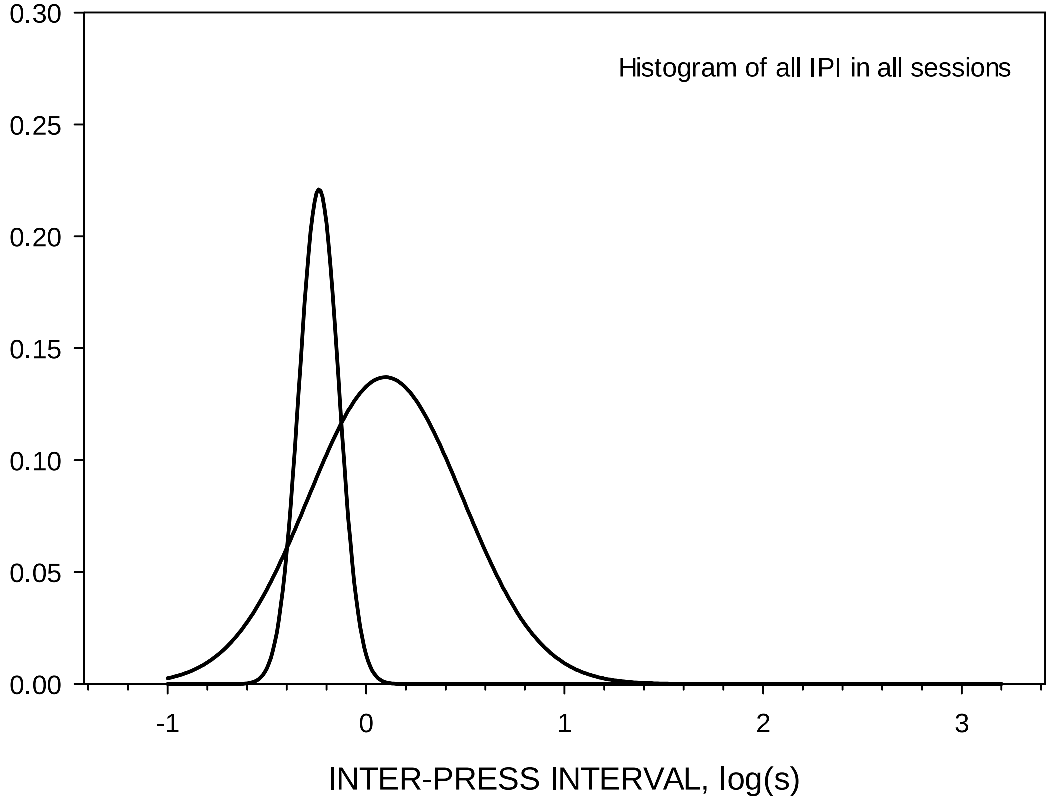

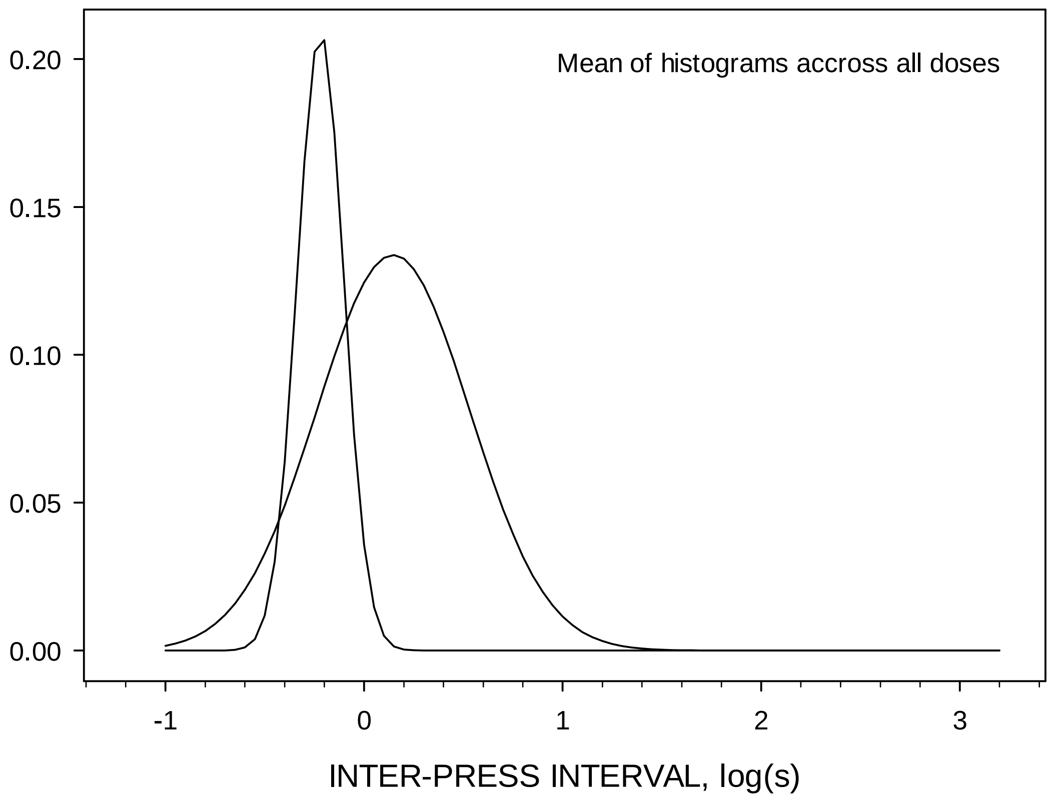

RAT 393

Baseline FR1

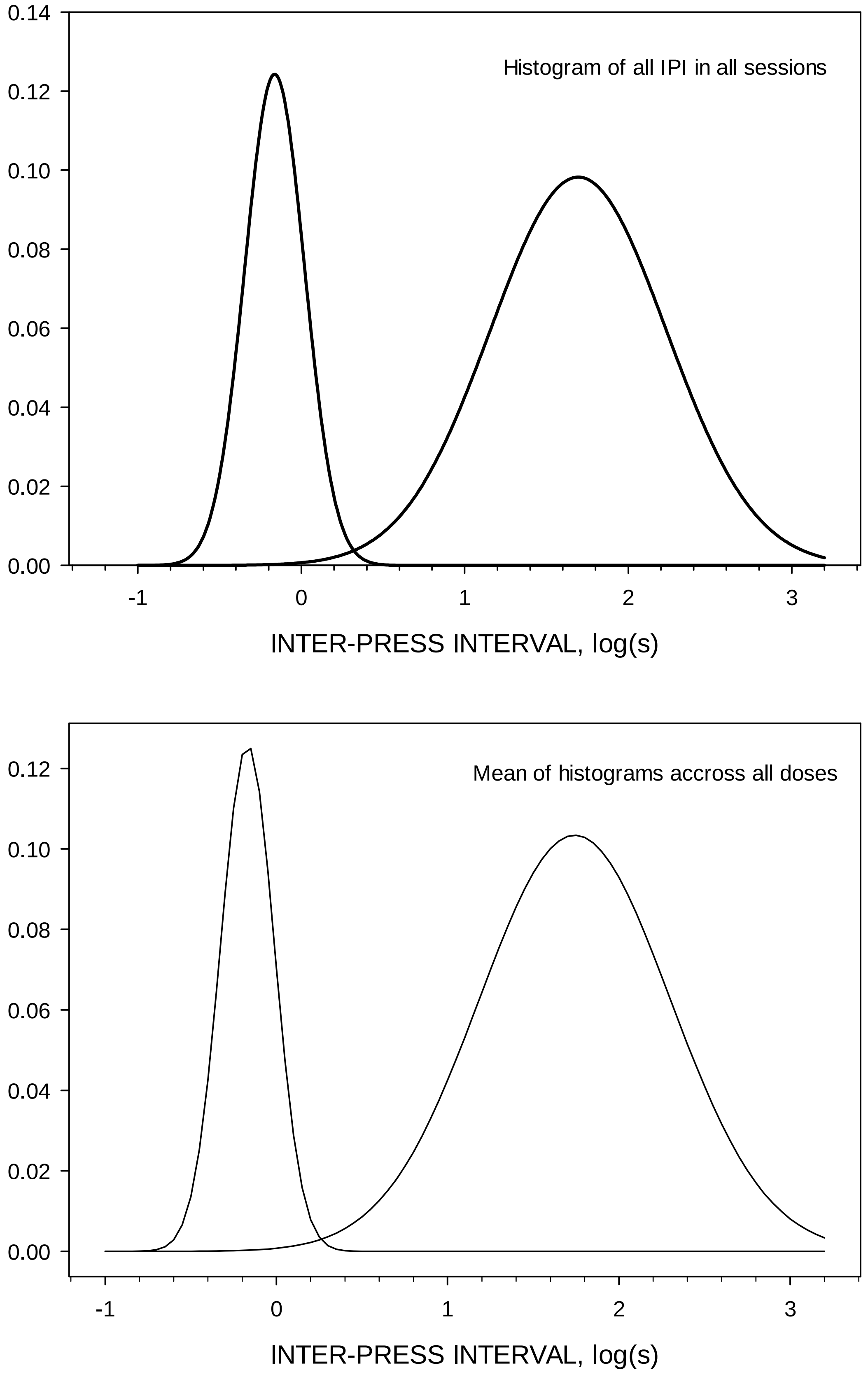

RAT 393

Post PR FR1

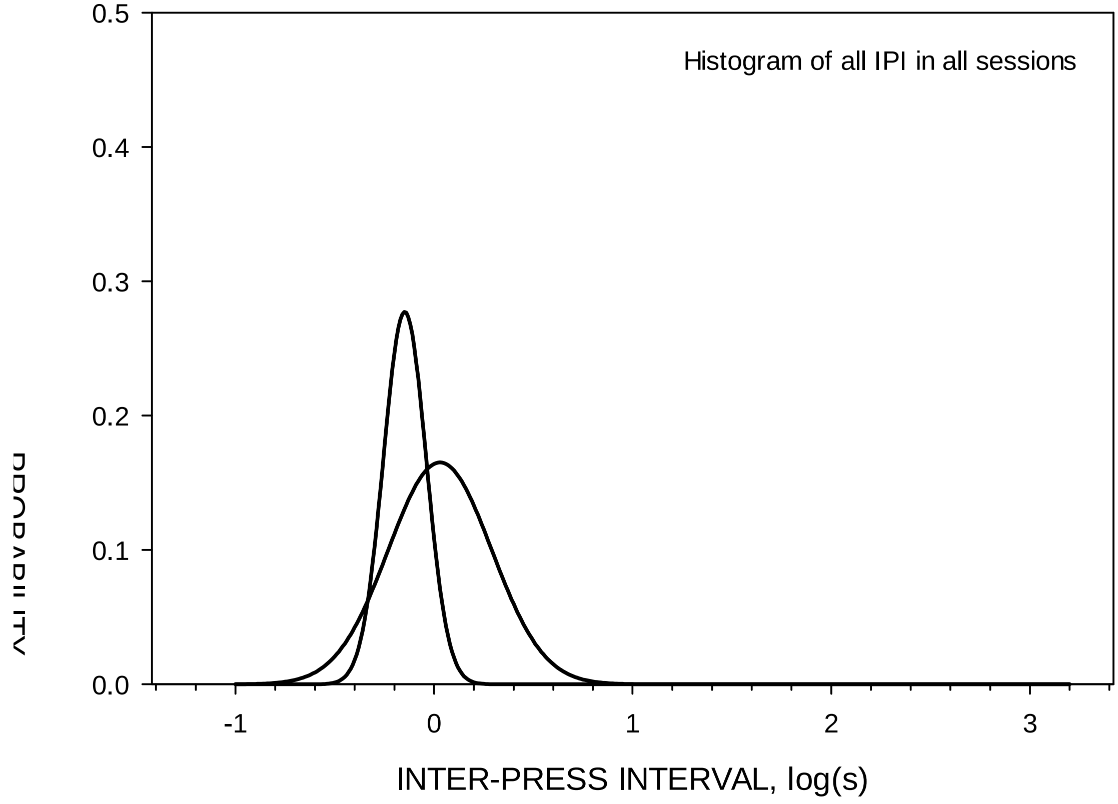

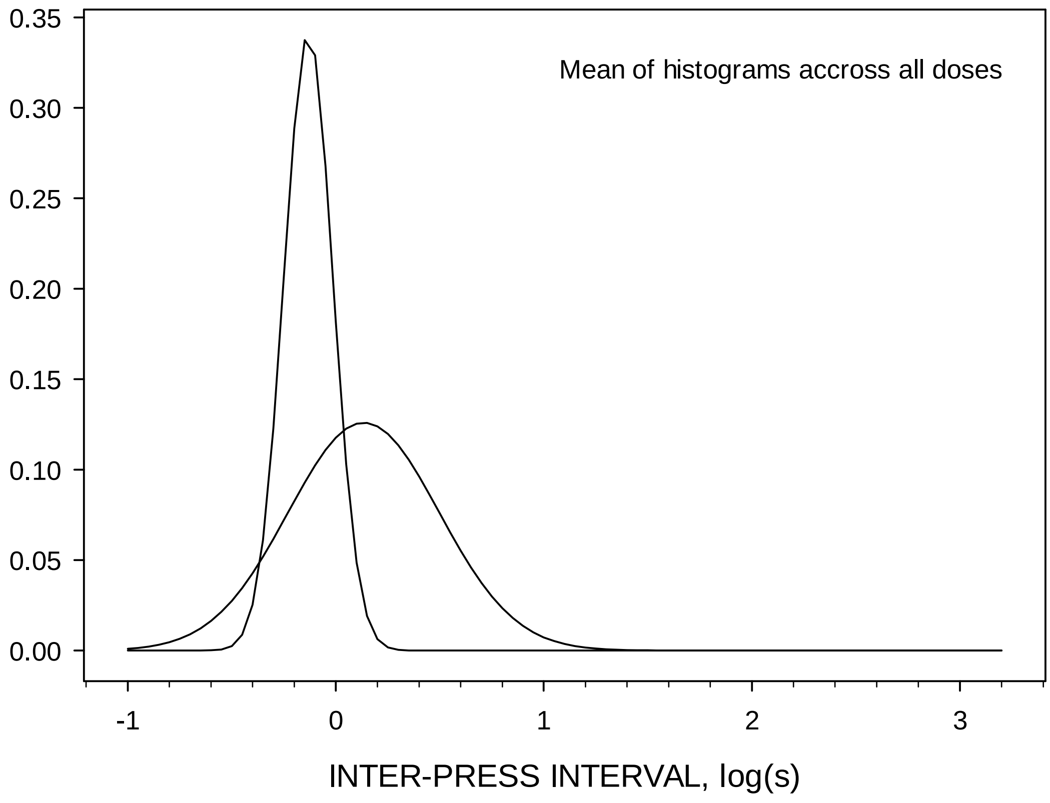

RAT 395

Baseline FR1

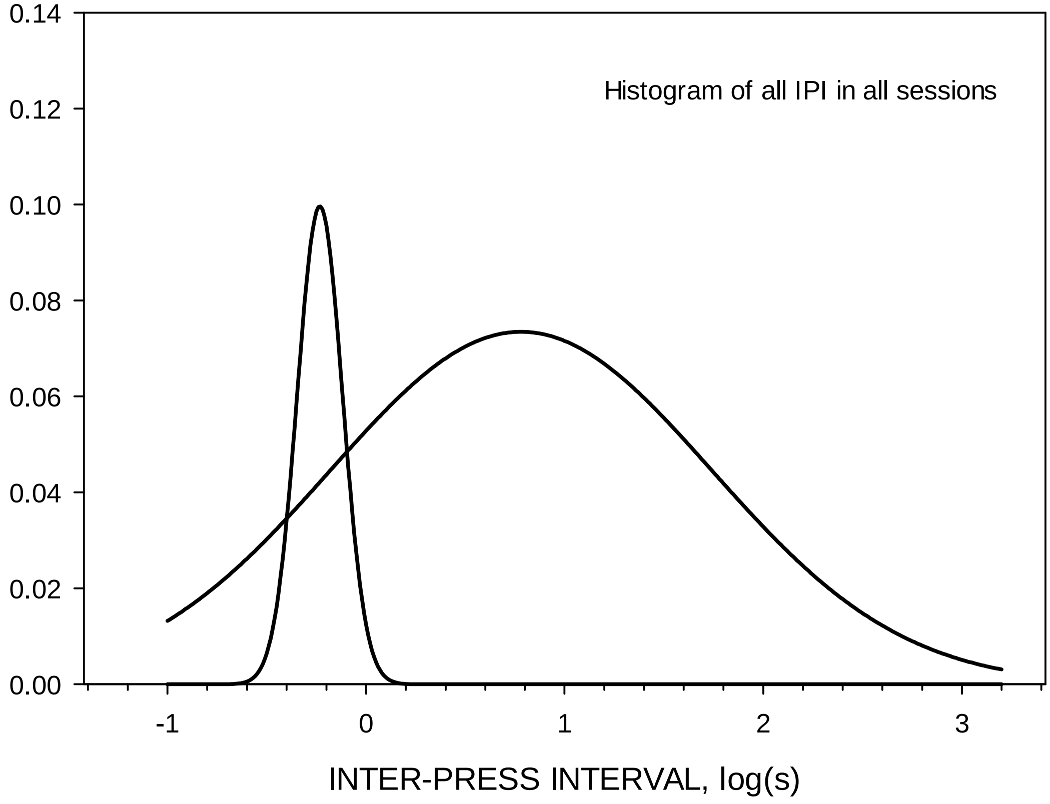

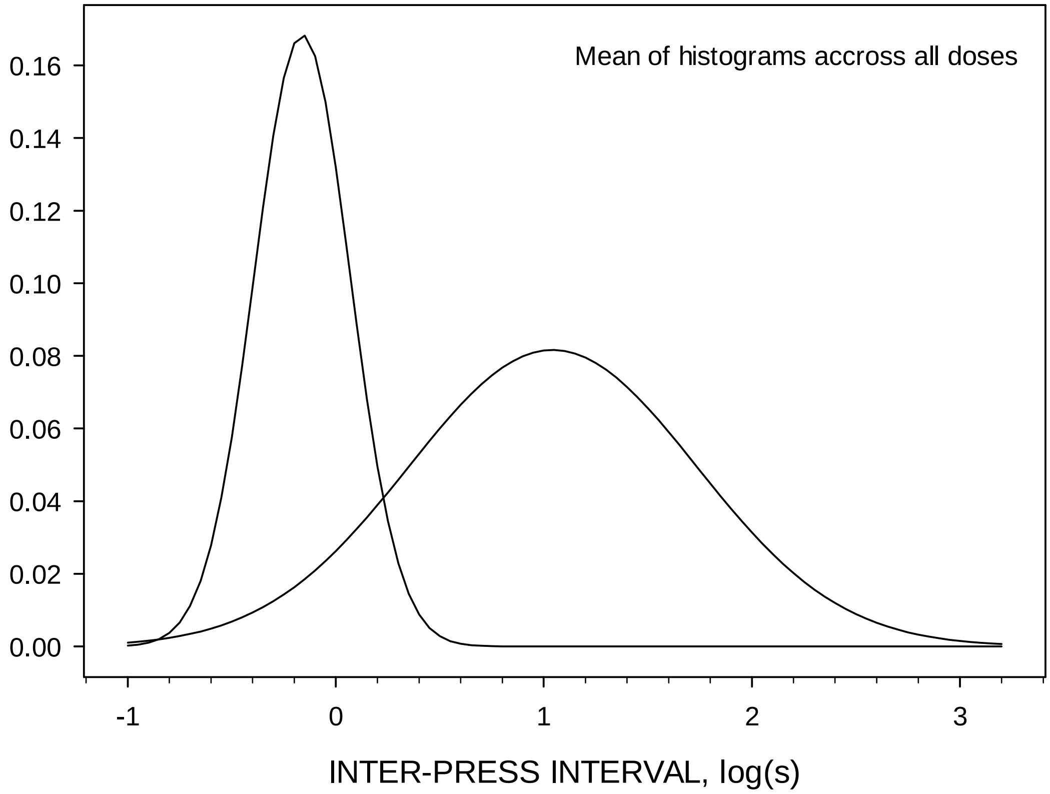

RAT 395

Post PR FR1

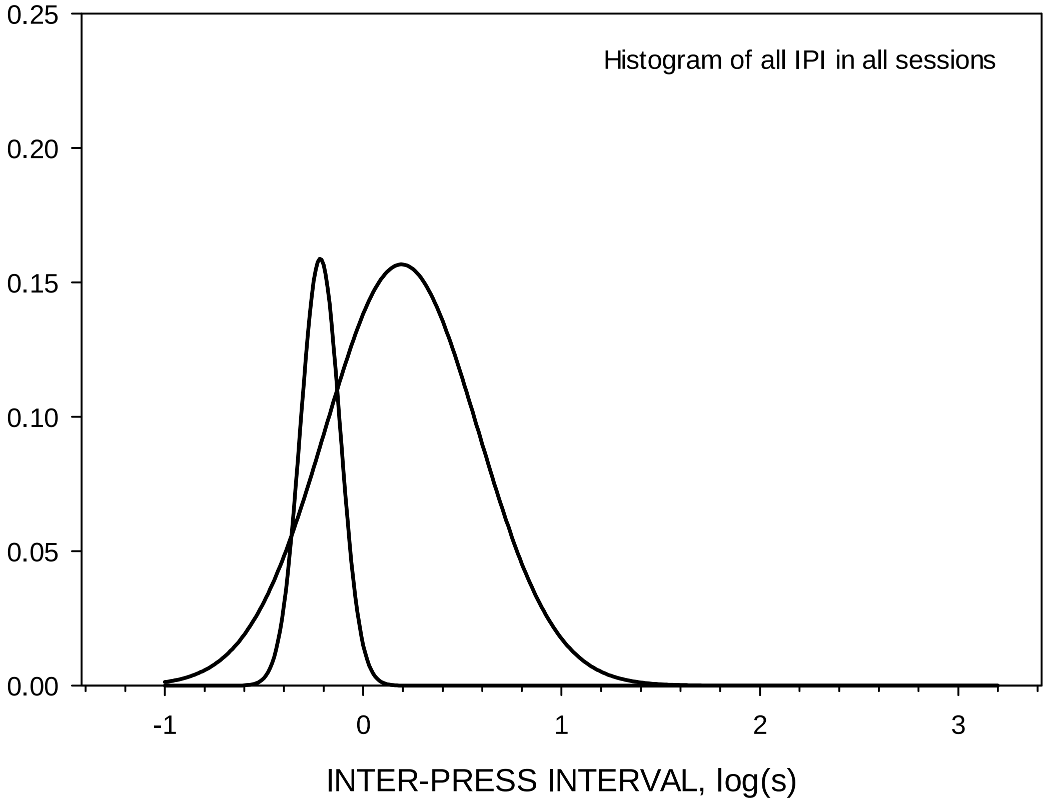

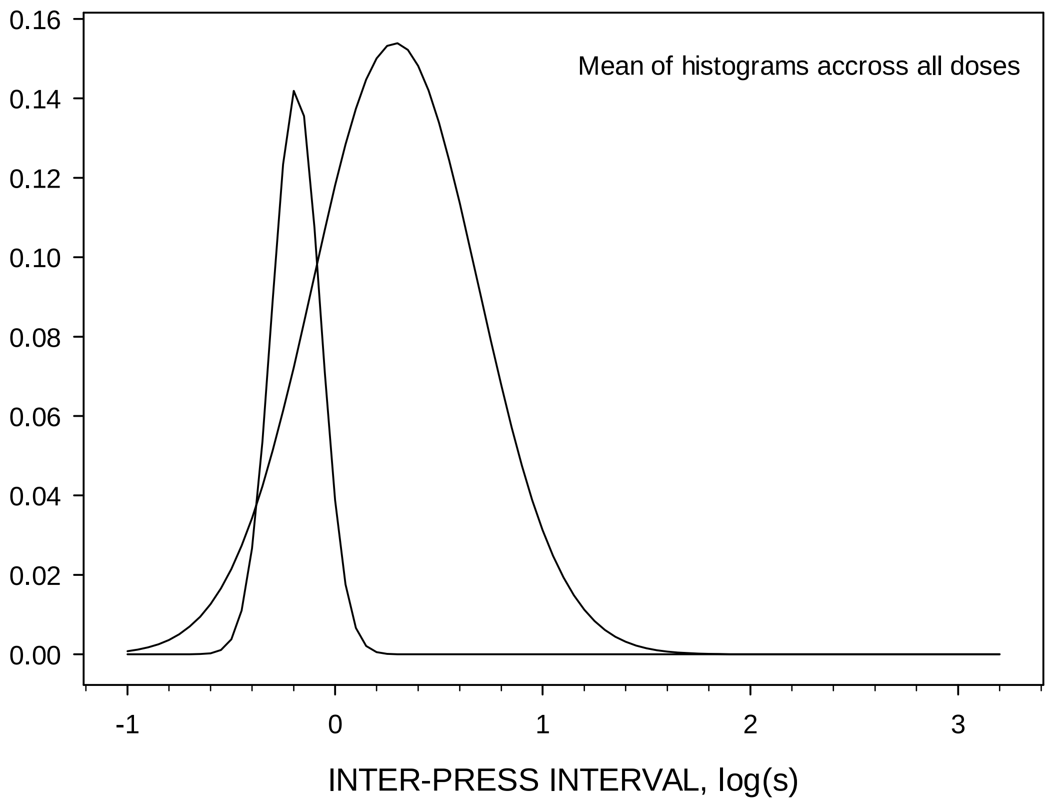

RAT 397

Baseline FR1

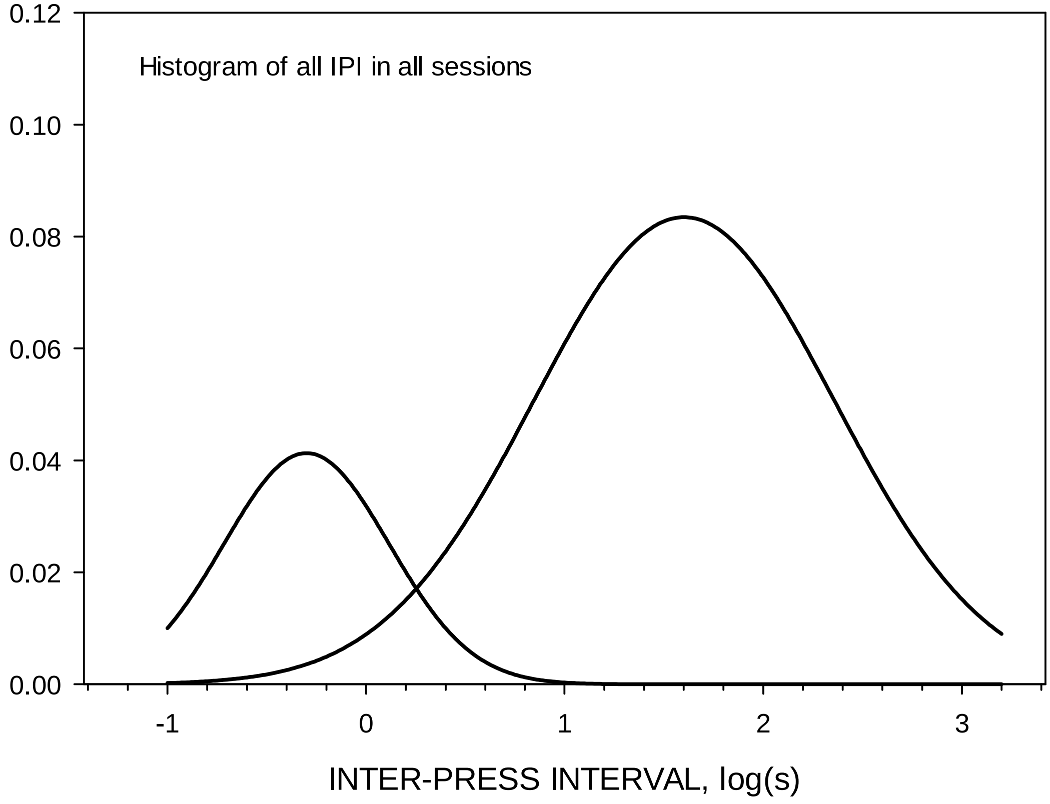

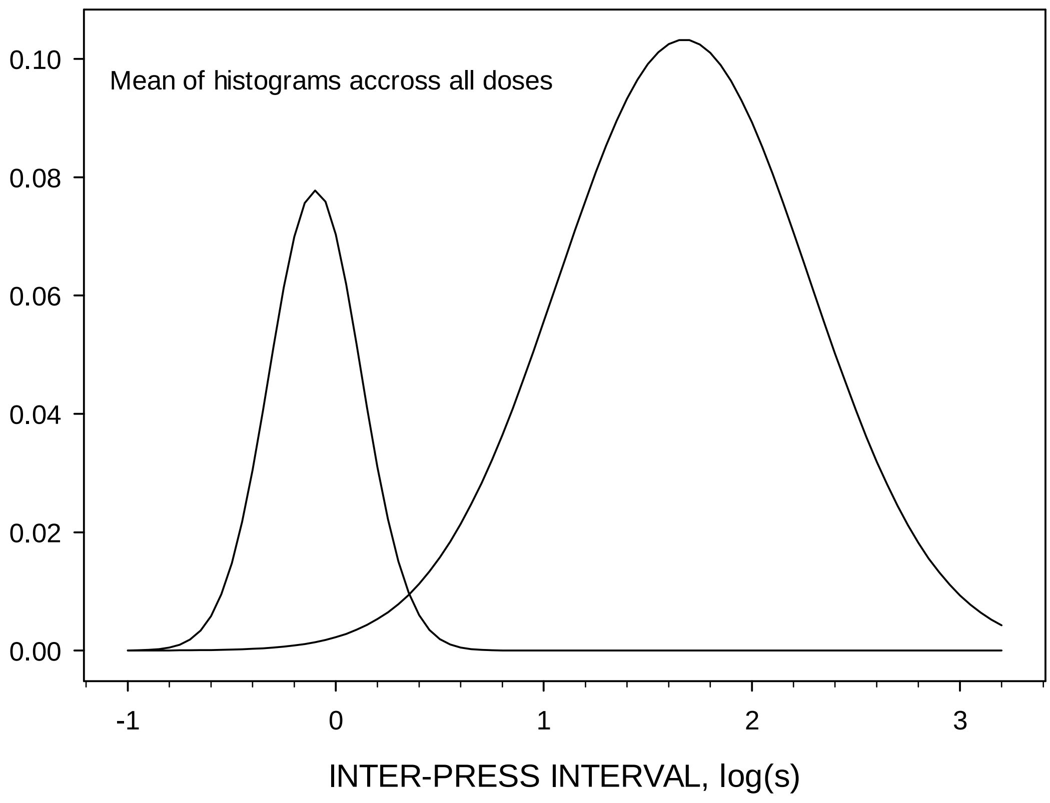

RAT 397

Post PR FR1

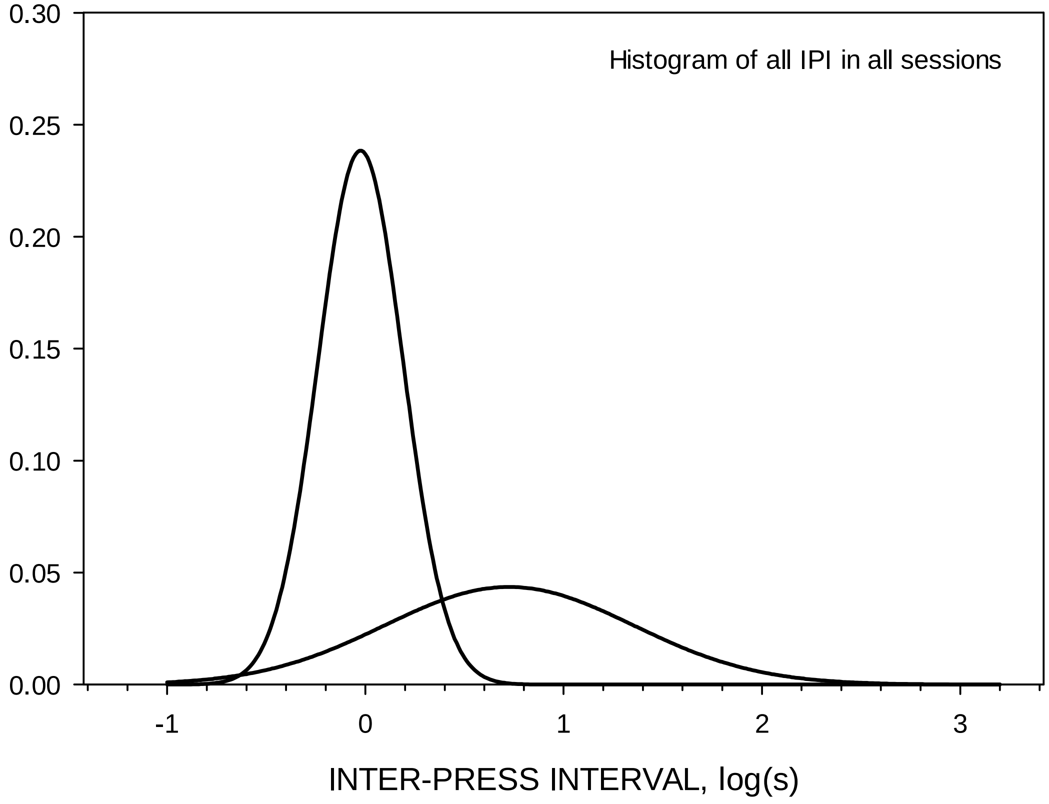

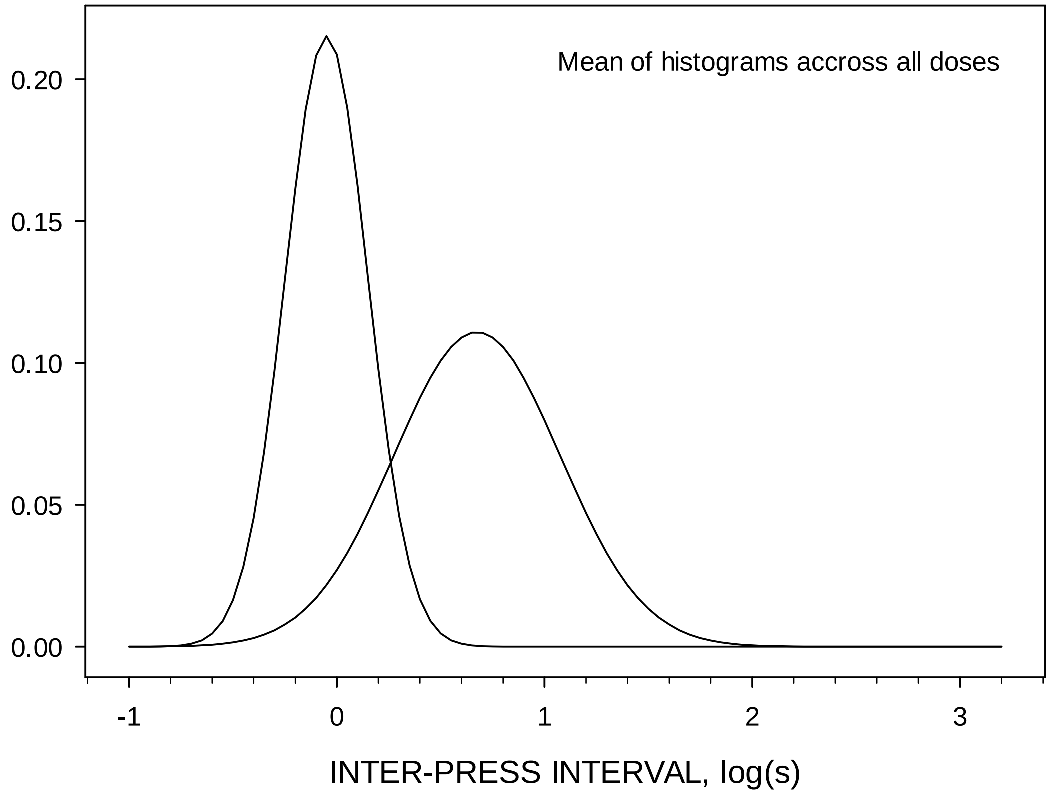

RAT 400

Baseline FR1

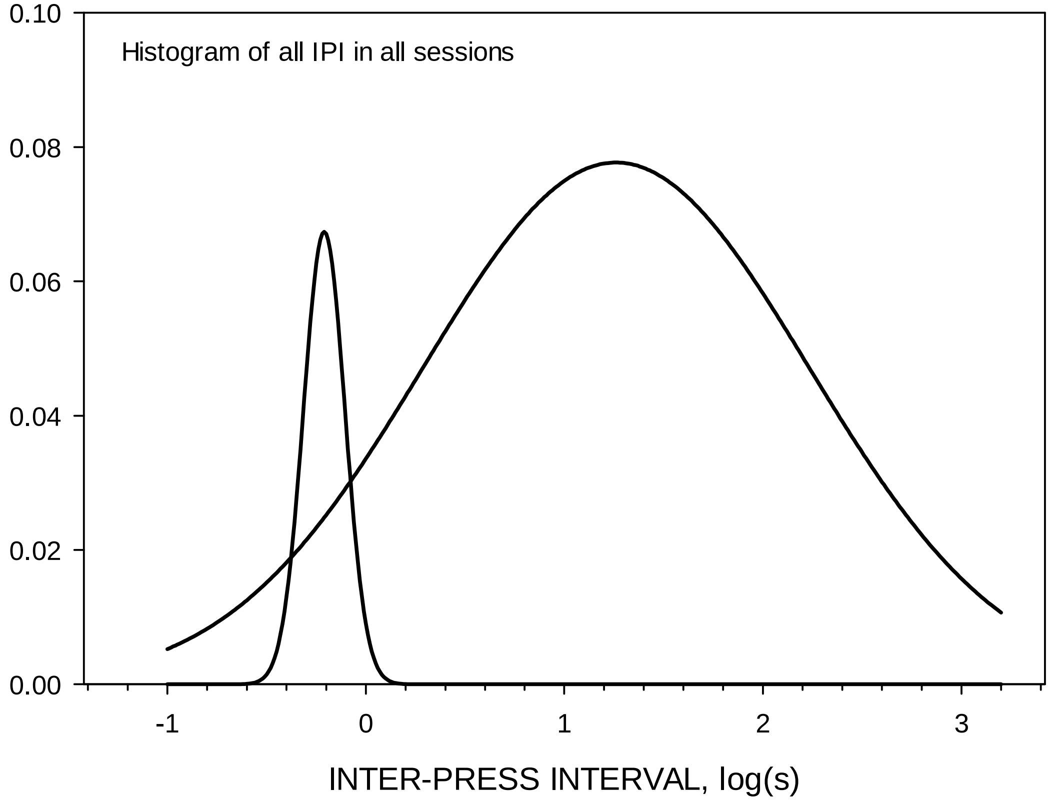

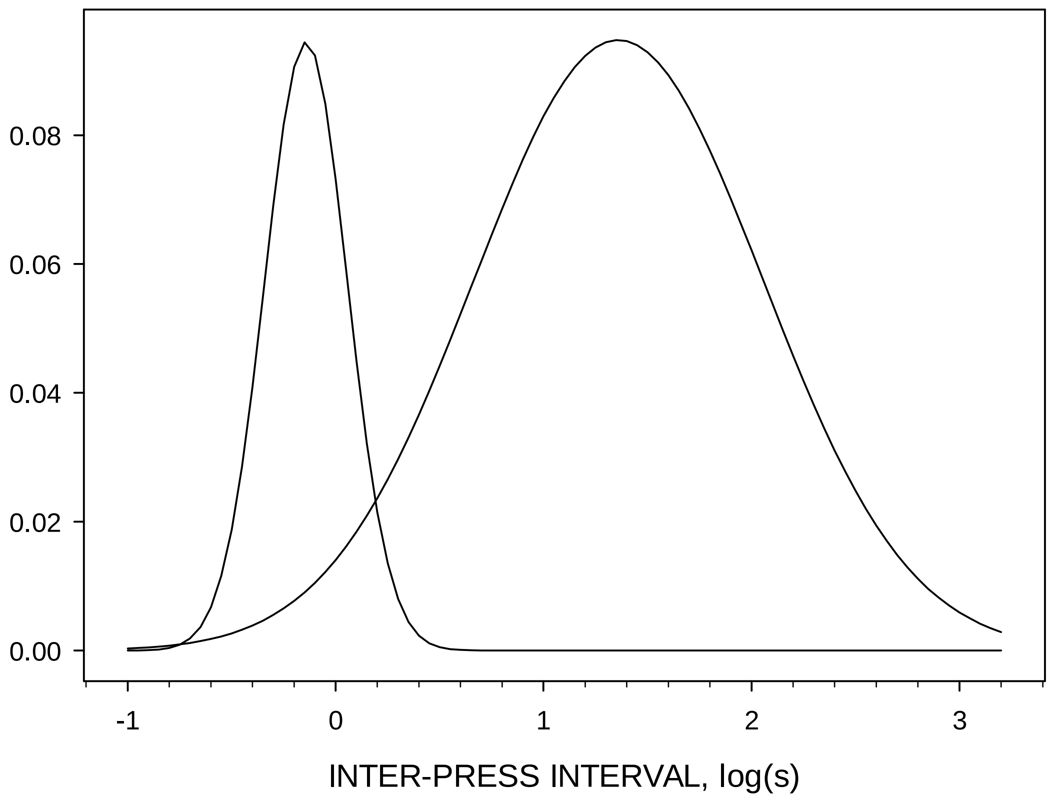

RAT 400

Post PR FR1

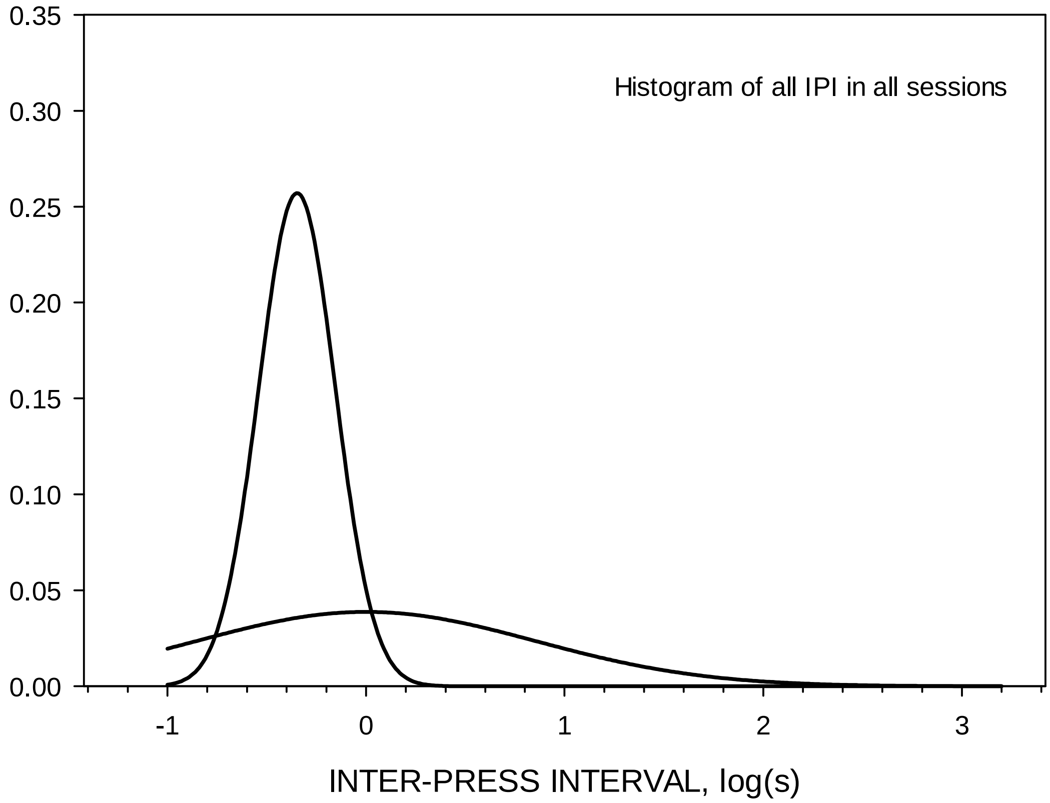

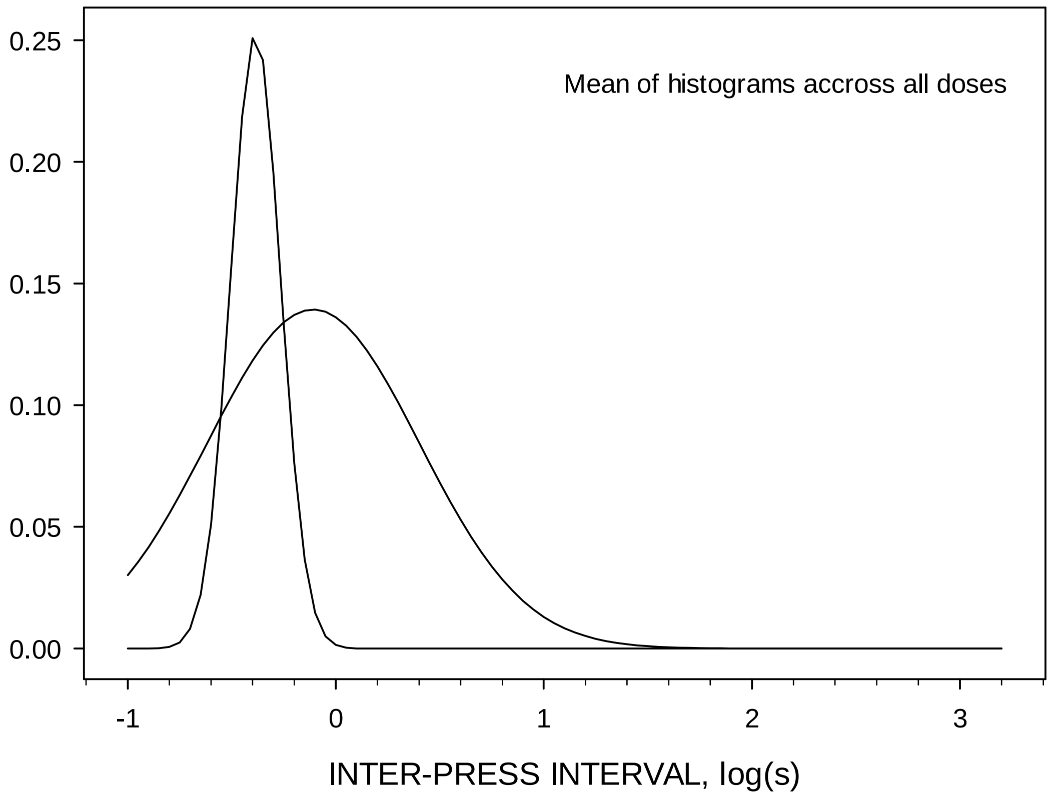

Supplement: Supplementary file 1 [file 41598_2021_98313_MOESM2_ESM.docx]
